# Nasopharyngeal carriage rates and serotype distribution of *Streptococcus pneumoniae* among school children with acute otitis media in Central Java, Indonesia

**DOI:** 10.1099/acmi.0.000249

**Published:** 2021-07-22

**Authors:** Daniel Joko Wahyono, Mifathuddin Majid Khoeri, Anton Budhi Darmawan, Siwi Pramatama Mars Wijayanti, Aris Mumpuni, Gita Nawangtantri, Wahyu Dwi Kusdaryanto, Korrie Salsabila, Dodi Safari

**Affiliations:** ^1^​ Faculty of Biology, Jenderal Soedirman University, Purwokerto, Indonesia; ^2^​ Eijkman Institute for Molecular Biology, Jakarta, Indonesia; ^3^​ Department of Otorhinolaryngology, Head and Neck Surgery, Faculty of Medicine, Jenderal Soedirman University, Purwokerto, Indonesia; ^4^​ Department of Public Health, Faculty of Health Sciences, Jenderal Soedirman University, Purwokerto, Indonesia; ^5^​ Faculty of Medicine, Jenderal Soedirman University, Purwokerto, Indonesia

**Keywords:** acute otitis media, nasopharyngeal carriage, serotype, *Streptococcus pneumoniae*

## Abstract

*Streptococcus pneumoniae* is a common bacterial pathogen that causes acute otitis media (AOM) in children. In this study, we investigated nasopharyngeal carriage rates and serotype distributions of *S. pneumoniae* among school children with AOM in Banyumas Regency, Central Java, Indonesia, from 2018 to 2019. Nasopharyngeal swab specimens and demographic data were collected from 122 children between the ages of 6 and 12. The specimens were cultured for the identification of *S. pneumoniae*, and serotyping was performed using a sequential multiplex PCR assay. We found that the *S. pneumoniae* carriage rate was 73 % (89/122) among children with AOM. Serotypes 23A (11 %) and 6A/6B (10 %) were the most common serotypes among the 91 cultured *S. pneumoniae* strains, followed by 3 (8 %), 14 (7 %), 6C/6D (7 %), 11A/11D (6 %), 15B/15C (4 %) and 35 B (4 %). Moreover, 41 % of the strains could be covered by the 13-valent pneumococcal conjugate vaccine, PCV13. In conclusion, high nasopharyngeal carriage rates of *S. pneumoniae* were found in school children with AOM, with almost half of the strains being the vaccine-type. This finding provides a baseline for nasopharyngeal carriage of *S. pneumoniae* in school children with AOM and supports the implementation of pneumococcal conjugate vaccines in Indonesia.

## Introduction

Otitis media, an infection of the middle ear, is a spectrum of diseases, including acute otitis media (AOM), chronic suppurative otitis media and otitis media with effusion [1, 2]. Otitis media is correlated with hearing impairment and long-term sequelae, especially in patients from low-income countries [1]. AOM is one of the most common infections in infancy and childhood, being the third main reason for antibiotic prescription in this age group [2]. The disease is characterized by purulent tympanic discharge and bulging of the tympanic membrane [3]. *Streptococcus pneumoniae* and *Haemophilus influenzae* are the predominant otopathogens associated with otitis media [4, 5]. *S. pneumoniae* causes acute AOM, which accounts for 30–50 % of all cases worldwide [6]. This bacterium is also the main cause of recurrent infections of otitis media [6]. Being an opportunistic pathogen, *S. pneumoniae* colonizes the mucosal surface of the human upper respiratory tract as normal human flora [7]. However, dissemination of this pathogen from the nasopharynx niche can lead to invasive diseases, such as pneumonia, meningitis and otitis media [7]. Colonization in the nasopharynx is associated with an increased risk of AOM [6, 8, 9]. Pneumococcal disease is preceded by bacterial invasion of the nasopharynx, which usually occurs without any noticeable symptoms. After establishing themselves in the nasopharynx of the host, the bacteria may spread via aerosolized droplets and colonize themselves in a new host. As a result, the data on nasopharyngeal carriage are also essential when developing prevention strategies against pneumococcal disease in the population [10].

The persistence of nasopharyngeal carriage of *S. pneumoniae* has been suggested as a risk factor for subsequent recurrent infections [8]. The *S. pneumoniae* strains isolated from middle-ear fluid specimens exhibit significant concordance with the strains isolated from nasopharyngeal/oropharyngeal specimens of children with acute otitis media, thereby indicating the high risk of persistent colonization of the bacteria in the affected individuals even after treatment [8]. It has been reported that aboriginal children vaccinated with the 13-valent pneumococcal conjugate vaccine, PCV13, had less nasopharyngeal carriage of combined *S. pneumoniae* and non-typeable *H. influenzae* (NTHi), but exhibited more polymicrobial (*S. pneumoniae* and NTHi) infections, which were identified from the middle-ear cultures [11]. Syrjänen *et al*. reported that the *S. pneumoniae* AOM occurred 3.5 times more frequently with newly acquired *S. pneumoniae* carriage [12]. The proportion of nasopharyngeal carriage of *S. pneumoniae* was high in the presence of AOM, particularly in *S. pneumoniae* AOM, with the most frequent nasopharyngeal serotypes among Finnish children being 6B, 6A, 11, 19F and 23F [13]. The majority of *S. pneumoniae* strains isolated from nasopharyngeal specimens obtained at the time of diagnosis of subsequent episodes of AOM were serotype identical at the completion of successful antibiotic treatment [14]. It is important to note that not all serotypes of *S. pneumoniae* cause AOM. There is an association between certain serotypes and nasopharyngeal colonization and their tendency to cause AOM. Therefore, it is crucial to understand the serotype distribution of *S. pneumoniae* in the affected regions. Furthermore, serotypes 3, 6A, 6B, 9V, 14, 19A, 19F and 23F are the most common pneumococcal serotypes that cause AOM worldwide [10]. However, non-vaccine serotypes, such as 11A and 15B/15C, are also known to frequently cause AOM worldwide [15]. Recently, it was reported that non-PCV13 serotypes (23A and 15B/C) were common in children under 5 years old with otitis media and those without in Taiwan [16]. Meanwhile, 19A remained the second most comment serotype among children with otitis media in Taiwan [16].

In Indonesia, the prevalence of chronic suppurative otitis media among school children was reported to be higher in rural areas (2.7%) than urban (0.7%) [17]. Recently, Anggraeni *et al*. reported that otitis media contributed to 57 % of all cases of hearing loss in school children in urban and rural sub-districts of Indonesia [18]. Our previous studies revealed a high prevalence rate (4.64 %) of AOM in the Banyumas Regency of Indonesia, where rates of household firewood use, low nutritional status and a family history of ear infections further increased the risk of acquiring AOM [19]. This demonstrates the importance of addressing AOM-related health issues. Currently, there are limited data on nasopharyngeal carriage of *S. pneumoniae* in children with AOM in Indonesia. Since 2017, Indonesia has implemented a regional PCV immunization programme in selected regions [20]. In general, the nasopharyngeal carriage rate of *S. pneumoniae* is approximately 43–55 % in Indonesian children [20–25]. In this study, we investigated nasopharyngeal carriage rates and serotype distributions of *S. pneumoniae* among school children with AOM in the Banyumas Regency, Central Java, Indonesia during the period of 2018–2019, prior to the introduction of PCV. This provides baseline data on the prevalence and serotype distribution of *S. pneumoniae* for pneumococcal vaccine implementation in the Indonesia population.

## Methods

### Study design and specimen collection

We conducted a cross-sectional study from 2018 to 2019 in the Banyumas Regency, Southwest of Central Java Province, Indonesia. This study was conducted at primary schools in six different regions of Indonesia, with more than 200 students in each school [19]. In total, 3574 school children were screened for AOM by medical doctors and otolaryngologists; 166 children were diagnosed with positive cases of AOM (4.64 %), as previously reported [19]. Nasopharyngeal swab specimens were collected from 122 school children with AOM using flocked nylon swabs (Cat. No. 503CS01), placed into 1 ml skim milk-tryptone-glucose-glycerol (STGG) medium, and stored in an icebox [22]. In study, the nasopharyngeal swab specimens were not available from 44 children with AOM. Then, the nasopharyngeal swab specimens were transported to the Faculty of Biology, Jenderal Soedirman University (Purwokerto, Central Java, Indonesia). The specimens were vortexed and stored at −80 °C within 4 h after collection. The specimens were also regularly shipped to the Eijkman Institute for Molecular Biology (Jakarta, Indonesia) on dry ice for the identification of *S. pneumoniae*. The detailed protocols are available at protocol.io (dx.doi.org/10.17504/protocols.io.bkuakwse).

### *S. pneumoniae* culture and identification

The swab specimens were enriched by transferring 100 µl of swab-STGG medium into 6 ml enrichment media consisting of 5 ml Todd-Hewitt broth with 0.5 % yeast extract and 1 ml rabbit serum, and then incubated in 5 % carbon dioxide (CO_2_) atmosphere at 37 °C for 5–6 h [26]. Then, 10 µl of enriched specimens were plated onto a blood agar plate and incubated in 5 % CO_2_ at 37 °C for 18–20 h. The plates were then examined to identify colonies exhibiting alpha-haemolysis and having a capsulated appearance. Suspected colonies were then sub-cultured on a blood agar plate for optochin susceptibility testing by placing an optochin disc (30 µg) on streaked agar. A bile solubility test was also performed to confirm optochin susceptibility. All isolates were stored in STGG media at −80 °C for further analysis. DNA extraction was performed as described previously [27]. Briefly, the *S. pneumoniae* cell suspension was heated at 100 °C for 5 min and immediately incubated at −20 °C for 5 min. The lysates were centrifuged at 1300 ***g*** for 10 min. The extracted DNA was stored at −20 °C until further use.

Serotype determination was performed by sequential multiplex conventional PCR, targeting the wzy gene (a component of the pneumococcal capsular locus) and consisted of eight reactions as described previously [27, 28]. A master mix for each reaction (25 µl) was made by adding PCR H_2_O, 0.5 µl of 5×PCR buffer (Promega), 3.5 µl of 25 mm magnesium chloride (MgCl_2_), 1 µl of 5 mm dNTPs (Promega), varied volumes of 25 µm forward primer and 25 µm reverse primer, 0.2 µl Taq Polymerase 2U, and 2.5 µl DNA template into a 1.5 ml microcentrifuge tube. For serotyping, conventional PCR was run under the following conditions: pre-denaturation at 94 °C for 4 min, followed by 30 cycles of denaturation at 94 °C for 45 s, annealing at 54 °C for 45 s and extension at 65 °C for 2.5 h. The DNA fragments were separated by electrophoresis and the DNA bands were visualized using a GelDoc machine. The serotype-negative isolates were confirmed by real-time PCR targeting *lytA* to confirm that the isolates were *S. pneumoniae*. Isolates with positive *lytA* were assigned as non-typeable *S. pneumoniae* by PCR [28, 29].

## Results

In this study, *S. pneumoniae* isolates were cultured and identified from 89 of 122 nasopharyngeal specimens (73 %) collected from primary school children with AOM between the ages of 6 and 12 in the Banyumas Regency, Central Java, Indonesia. We confirmed that all the *S. pneumoniae* strains were susceptible to optochin and positive for the *lytA* gene by PCR. The participant characteristics are shown in Table 1. We observed that the nasopharyngeal carriage rate of *S. pneumoniae* was higher in males (80 %) than in females (67 %) (Table 1). We found that the carriage rate of *S. pneumoniae* was higher in children from the family with lower income status than normal/high income status (85 % vs 65 %). We also observed that children aged 6 to 7 exhibited higher colonization by *S. pneumoniae* (79 %) than those older than 7 (Table 1).

**Table 1.** Participants characteristics related to pneumococcal carriage

Variable		No. tested (*n*)	No. with *S. pneumoniae* detected (*n*)	Prevalence (%)
Overall		122	89	73
Age (year)				
	6–7	33	26	79
	8–9	49	38	78
	10–12	40	25	63
Sex				
	Male	59	47	80
	Female	63	42	67
BMI profile				
	Under weight	26	19	73
	Healthy weight	72	52	72
	Over weight	12	10	83
	Obesity	12	8	67
Source of cooking				
	Gas	92	71	77
	Wood	26	20	77
	Gasoline	3	3	100
No. of family members				
	1–5	104	81	78
	> 5	18	14	78
Income status				
	Low income	74	61	82
	High income	48	31	65

*BMI (Body Mass Index) profile was calculated based on BMI Percentile Calculator for Child and Teen (https://www.cdc.gov/healthyweight/bmi/calculator.html).

**Fig. 1.**
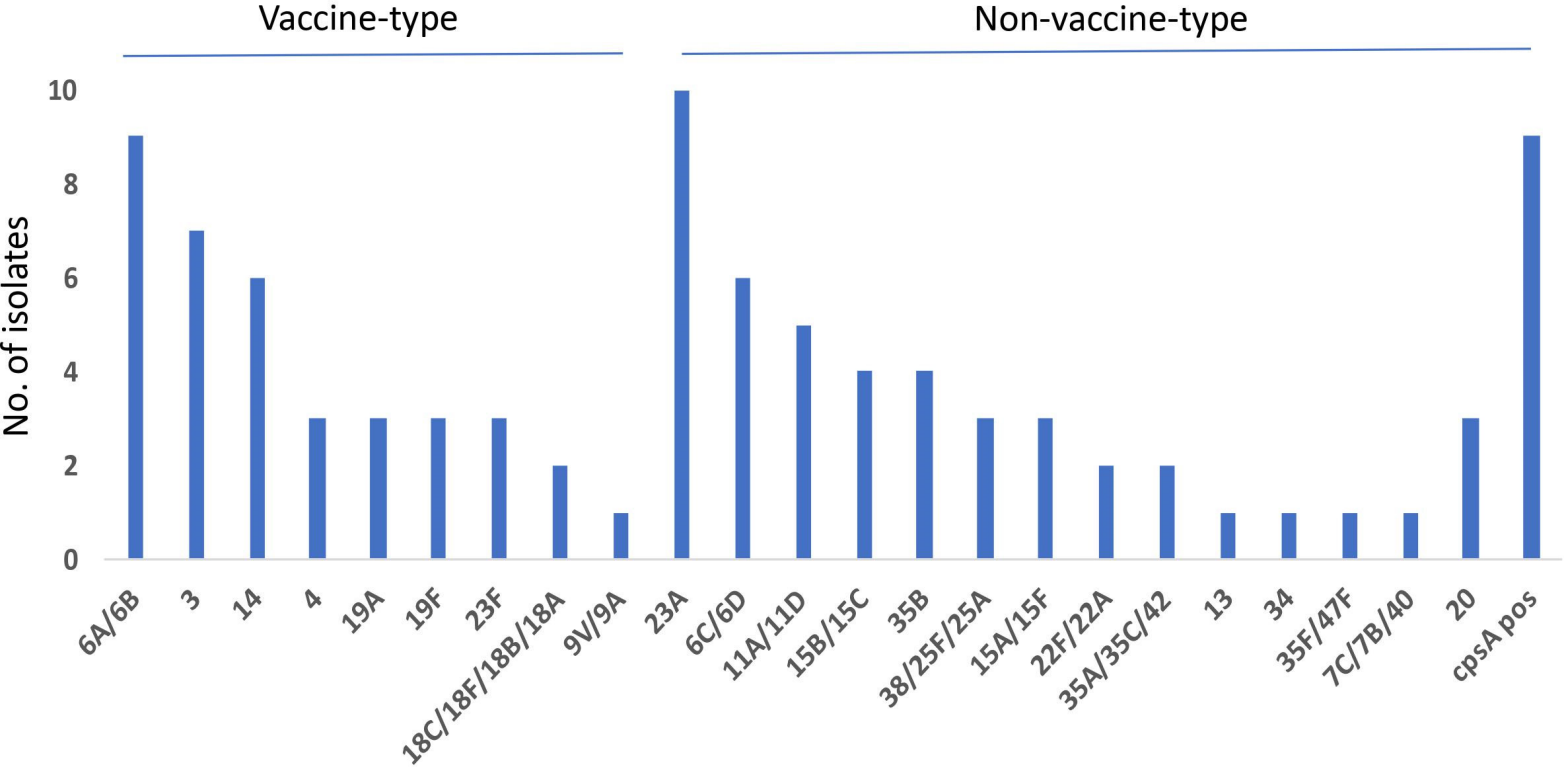
Serotype distribution of *S. pneumoniae* isolates among 91 *S. pneumoniae* carriage isolates of school children in Banyumas regency, Central Java, Indonesia.

In this study, we found multiple serotypes of pneumococcal isolates from two children, one having serotype 3 with serotype 23A and the other having serotype 23F with serotype 6C/6D. Among the 24 identified *S. pneumoniae* serotypes, we found that the most frequent serotypes were 23A (*n*=10, 11 %) and 6A/6B (*n*=9, 10 %), followed by 3 (*n*=7, 8 %), 14 (*n*=6, 7 %), 6C/6D (*n*=6, 7 %), 11A/11D (*n*=5, 6 %), 15B/15C (*n*=4, 4 %), 35 B (*n*=4, 4 %), 4 (*n*=3, 3 %), 20 (*n*=3, 3 %), 19A (*n*=3, 3 %), 19F (*n*=3, 3 %), 23F (*n*=3, 3 %), 38/25F/25A (*n*=3, 3 %), 15A/15F (*n*=2, 2 %), sg18 (*n*=2, 2 %), 22F/22A (*n*=2, 2 %), 35A/35C/42 (*n*=2, 2 %), 13 (*n*=1, 1 %), 34 (*n*=1, 1 %), 35F/47F (*n*=1, 1 %), 7C/7B/40 (*n*=1, 1 %) and 9V/9A (*n*=1, 1 %) (Fig. 1). We determined nine *S. pneumoniae* isolates (10 %) to be non-typeable with *cpsA* gene positivity using PCR. Among the 91 *S*. *pneumoniae* isolates, 41 % (*n*=37) were serotypes included in the PCV13 vaccine with 6A/6B (*n*=9) being the most common serotype, followed by 3 (*n*=3), 14 (*n*=6), 4 (*n*=3), 19A(*n*=3), 19F (*n*=3), sg18 (*n*=2) and 23F (*n*=3) serotypes. In this study, we identified that 23A (*n*=10) was the most common serotype not included in the current pneumococcal conjugate vaccine, followed by the 6C/6D (*n*=6) and 11A/11D (*n*=5) serotypes.

## Discussion

Nasopharyngeal carriage of *S. pneumoniae* has been associated with an increased risk of otitis media [6]. Previously, there was significant concordance between *S. pneumoniae* isolated from middle-ear fluid specimens and the nasopharynx/oropharynx colonization in children with AOM between 1 and 16 years of age [8]. Otitis-prone children were reported to have greater colonization of *S. pneumoniae* in the nasopharynx than non-otitis-prone children, indicating that colonization by *S. pneumoniae* might lead to the development of otitis media. Furthermore, the presence of other bacteria, such as *H. influenzae*, *Moraxella catharralis* and *S. pyogenes*, as well as other viral respiratory pathogens causing microenvironment changes, including fever and immune response suppression, have also been reported as risk factors for AOM [30]. In this study, we found that nasopharyngeal carriage rates of *S. pneumoniae* were high in primary school children with AOM (73 %), between the ages of 6 and 12, in Indonesia. In general, the incidence of nasopharyngeal carriage among Indonesian children is 55 % (ranging from 31.2–84.5%) [20]. Another study in aboriginal children vaccinated with pneumococcal vaccines showed that nasopharyngeal carriage rates were high (approximately 77.9 %) in them, with more than half of the children exhibiting otitis media symptoms [11].

The high rate of *S. pneumoniae* nasopharyngeal carriage in children with AOM might be due to the disease being mostly caused by *S. pneumoniae*. Nasopharyngeal carriage is a prerequisite for pneumococcal diseases, such as AOM [31]. Bacterial AOM, which is more symptomatic, is generally predisposed to viral infections, such as those caused by the influenza viruses (A and B), rhinoviruses, respiratory syncytial virus and adenovirus [32]. Pneumococcus, which colonizes the nasopharynx asymptomatically, can sense nasopharyngeal environmental changes caused by viral infections by upregulating specific genes involved in the formation of biofilms, dissemination to other sites, and evasion of the host immune system [33]. One study found that almost 70 % of *S. pneumoniae* isolated from the nasopharynx of children with AOM produced biofilms and 33.5 % of them were strong biofilm producers [34]. Bacteria in biofilms are more resistant to the action of antimicrobials than planktonic forms [35].

The nasopharynx of children with AOM had significantly lower microbial diversity than the nasopharynx of healthy children, with the dominant species belonging to the genera *Haemophilus*, *Moraxella*, *Streptococcus*, *Corynebacterium* and *Alloicoccus*. The microbiome during AOM was similar to that of the middle-ear fluid microbiome at the onset of AOM. *Haemophilus*, *Moraxella* and *Streptococcus* constitute the most abundant microbiota in both healthy and AOM-affected children [36]. This indicates that AOM is a consequence of the otopathogens ascending from the nasopharynx to the middle ear. Moreover, bacterial richness increased with age in children with AOM between the ages of 0 and 6. Members of the *Moraxellaceae* family were found to be the most abundant during the first year of life. Meanwhile, the abundance of *Streptococcaceae* peaked at around 2–3 years of age [37].

In this study, we discovered that PCV13 provided a vaccine coverage of 40.7 %, with 6A/6B being the most common serotype, followed by 3, 14, 4, 19A, 19F, sg18 and 23F serotypes. We also found that 23A was the most common serotype not included in the current pneumococcal conjugate vaccine, followed by the 6C/6D and 11A/11D serotypes. Serotype distribution may vary across different regions in Indonesia [21, 24, 38]. Another study in Iceland showed that the proportion of vaccine-type pneumococcal strains recovered from the middle-ear specimens was significantly decreased, while the proportion of non-vaccine serotype (6C, 15B/C, 23A and 23 B) strains was increased post-PCV introduction, with most of the serotype 6 C *S*. *pneumoniae* strains being reported as multidrug-resistant strains [39]. PCV13 lowered the carriage rate of *S. pneumoniae* due to the significant reduction in the prevalence of vaccine serotypes, except for serotype 3. However, non-PCV13 serotypes 11, 15 and 23 (excluding 23F) were frequently found after PCV13 implementation [40]. In addition, another study involving Chilean children with otitis media showed that the serotype distribution among children with otitis media changed over time, with vaccine serotypes being the most prevalent [41]. In a report from Spain, a PCV7 vaccine was reported to decrease the prevalence of vaccine serotypes causing otitis media. However, there was an increase in the prevalence of otitis media caused by non-PCV7 serotypes that were not included in PCV10/13 (serotype 1, 5 7F, 3, 6A and 19A), with serotype 19A being the predominant serotype. The frequency of otitis media caused by non-vaccine serotypes has also been reported to increase over time after PCV7 vaccination [42].

One limitation of our study was that we could only collect nasopharyngeal specimens from children with AOM. We did not collect middle-ear fluid specimens due to the limited facilities available for the collection of middle-ear fluid. Therefore, we could not compare the bacteria from nasopharyngeal specimens with those from middle-ear fluid specimens. Therefore, similar carriage studies should be conducted across different regions in Indonesia to monitor the impact of the pneumococcal conjugate vaccine implementation. In conclusion, nasopharyngeal carriage rates of *S. pneumoniae* were reported to be high among Indonesian school children with AOM, with almost half of the identified strains being vaccine-serotype. The serotype distribution and prevalence of *S. pneumoniae* found in this study are important baseline data that could aid in the implementation of pneumococcal vaccine programmes in Indonesia. Furthermore, the results of our study will be important to monitor the impact of pneumococcal vaccines in Indonesian population.

## References

[1] Schilder AGM, Chonmaitree T, Cripps AW, Rosenfeld RM, Casselbrant ML (2016). Otitis media. Nat Rev Dis Primer.

[2] Danishyar A, Ashurst JV (2020). StatPearls. Treasure Island (FL).

[3] Thomas JP, Berner R, Zahnert T, Dazert S (2014). Acute otitis media--a structured approach. Dtsch Arztebl Int.

[4] Ngo CC, Massa HM, Thornton RB, Cripps AW (2016). Predominant bacteria detected from the middle ear fluid of children experiencing Otitis media: A systematic review. PLoS One.

[5] Levy C, Varon E, Ouldali N, Wollner A, Thollot F (2019). Bacterial causes of otitis media with spontaneous perforation of the tympanic membrane in the era of 13 valent pneumococcal conjugate vaccine. PLOS ONE.

[6] Bergenfelz C, Hakansson AP (2017). *Streptococcus pneumoniae* otitis media pathogenesis and how it informs our understanding of vaccine strategies. Curr Otorhinolaryngol Rep.

[7] Weiser JN, Ferreira DM, Paton JC (2018). *Streptococcus pneumoniae*: transmission, colonization and invasion. Nat Rev Microbiol.

[8] Korona-Glowniak I, Zychowski P, Siwiec R, Mazur E, Niedzielska G (2018). Resistant *Streptococcus pneumoniae* strains in children with acute otitis media– high risk of persistent colonization after treatment. BMC Infect Dis.

[9] Rupa V, Isaac R, Rebekah G, Manoharan A (2016). Association of *Streptococcus pneumoniae* nasopharyngeal colonization and other risk factors with acute otitis media in an unvaccinated Indian birth cohort. Epidemiol Infect.

[10] Rodgers GL, Arguedas A, Cohen R, Dagan R (2009). Global serotype distribution among *Streptococcus pneumoniae* isolates causing otitis media in children: Potential implications for pneumococcal conjugate vaccines. Vaccine.

[11] Leach AJ, Wigger C, Beissbarth J, Woltring D, Andrews R (2016). General health, otitis media, nasopharyngeal carriage and middle ear microbiology in Northern Territory Aboriginal children vaccinated during consecutive periods of 10-valent or 13-valent pneumococcal conjugate vaccines. Int J Pediatr Otorhinolaryngol.

[12] Syrjänen RK, Auranen KJ, Leino TM, Kilpi TM, Mäkelä PH (2005). Pneumococcal acute otitis media in relation to pneumococcal nasopharyngeal carriage. Pediatr Infect Dis J.

[13] Syrjänen RK, Kilpi TM, Kaijalainen TH, Herva EE, Takala AK (2001). Nasopharyngeal carriage of *Streptococcus pneumoniae* in Finnish children younger than 2 years old. J Infect Dis.

[14] Libson S, Dagan R, Greenberg D, Porat N, Trepler R (2005). Nasopharyngeal carriage of *Streptococcus pneumoniae* at the completion of successful antibiotic treatment of acute Otitis media predisposes to early clinical recurrence. J Infect Dis.

[15] Napolean M, Rosemol V, John M, Varghese AM, Periyasamy J (2021). Nasopharyngeal colonization of otopathogens in South Indian children with acute otitis media – A case control pilot study. J Otol.

[16] Chen CH, Chen C-L, Arguedas A, Southern J, Hsiao CC (2020). Divergent serotype distribution between children with Otitis media and those without in the pneumococcal conjugate vaccine era. J Microbiol Immunol Infect.

[17] Anggraeni R, Hartanto WW, Djelantik B, Ghanie A, Utama DS (2014). Otitis media in indonesian urban and rural school children. Pediatr Infect Dis J.

[18] Anggraeni R, Carosone-Link P, Djelantik B, Setiawan EP, Hartanto WW (2019). Otitis media related hearing loss in Indonesian school children. Int J Pediatr Otorhinolaryngol.

[19] Wijayanti SP, Wahyono DJ, Rejeki DSS, Octaviana D, Mumpuni A (2021). Risk factors for acute otitis media in primary school children: a case-control study in Central Java, Indonesia. J Public Health Res.

[20] Kartasasmita CB, Rezeki Hadinegoro S, Kurniati N, Triasih R, Halim C (2020). Epidemiology, nasopharyngeal carriage, serotype prevalence, and antibiotic resistance of *streptococcus pneumoniae* in Indonesia. Infect Dis Ther.

[21] Hadinegoro SR, Prayitno A, Khoeri MM, Djelantik IGG, Dewi NE (2016). Nasopharyngeal crriage of *Streptococcus pneumoniae* in healthy children under five years old in Central Lombok Regency, Indonesia. Southeast Asian J Trop Med Public Health.

[22] Safari D, Kurniati N, Waslia L, Khoeri MM, Putri T (2014). Serotype distribution and antibiotic susceptibility of *Streptococcus pneumoniae* strains carried by children infected with human immunodeficiency virus. PloS One.

[23] Muktiarti D, Khoeri MM, Tafroji W, Waslia L, Safari D (2021). Serotypes and Antibiotic Susceptibility Profile of Streptococcus Pneumoniae Isolated from Nasopharynges of Children Infected with Hiv in Jakarta, Indonesia, Pre- and Post-Pneumococcal Vaccination.

[24] Dunne EM, Murad C, Sudigdoadi S, Fadlyana E, Tarigan R (2018). Carriage of *Streptococcus pneumoniae*, *Haemophilus influenzae*, *Moraxella catarrhalis*, and *Staphylococcus aureus* in Indonesian children: A cross-sectional study. PloS One.

[25] Farida H, Severin JA, Gasem MH, Keuter M, Wahyono H (2014). Nasopharyngeal carriage of *Streptococcus pneumonia* in pneumonia-prone age groups in Semarang, Java Island, Indonesia. PloS One.

[26] Carvalho MDG, Pimenta FC, Jackson D, Roundtree A, Ahmad Y (2010). Revisiting pneumococcal carriage by use of broth enrichment and PCR techniques for enhanced detection of carriage and serotypes. J Clin Microbiol.

[27] Pai R, Gertz RE, Beall B (2006). Sequential multiplex PCR approach for determining capsular serotypes of *Streptococcus pneumoniae* isolates. J Clin Microbiol.

[28] Harimurti K, Saldi SRF, Dewiasty E, Alfarizi T, Dharmayuli M (2021). *Streptococcus pneumoniae* carriage and antibiotic susceptibility among Indonesian pilgrims during the Hajj pilgrimage in 2015. PLOS ONE.

[29] Carvalho M da GS, Tondella ML, McCaustland K, Weidlich L, McGee L (2007). Evaluation and improvement of real-time PCR assays targeting lytA, ply, and psaA genes for detection of pneumococcal DNA. J Clin Microbiol.

[30] Bergenfelz C, Hakansson AP (2017). *Streptococcus pneumoniae* otitis media pathogenesis and how it informs our understanding of vaccine strategies. Curr Otorhinolaryngol Rep.

[31] Mulholland K, Satzke C (2012). Serotype replacement after pneumococcal vaccination. The Lancet.

[32] Bakaletz LO (2010). Immunopathogenesis of polymicrobial otitis media. J Leukoc Biol.

[33] Bergenfelz C, Hakansson AP (2017). *Streptococcus pneumoniae* otitis media pathogenesis and how it informs our understanding of vaccine strategies. Curr Otorhinolaryngol Rep.

[34] Vermee Q, Cohen R, Hays C, Varon E, Bonacorsi S (2019). Biofilm production by *Haemophilus influenzae* and *Streptococcus pneumoniae* isolated from the nasopharynx of children with acute otitis media. BMC Infect Dis.

[35] Yadav MK, Chae SW, Song JJ (2012). In vitro *Streptococcus pneumoniae* biofilm formation and In vivo middle ear mucosal biofilm in a rat model of acute otitis induced by *S. pneumoniae*. Clin Exp Otorhinolaryngol.

[36] Xu L, Gill S, Xu L, Gonzalez E, Pichichero ME (2019). Comparative analysis of microbiome in nasopharynx and middle ear in young children with acute otitis media. Front Genet.

[37] Brugger SD, Kraemer JG, Qi W, Bomar L, Oppliger A (2019). Age-dependent dissimilarity of the nasopharyngeal and middle ear microbiota in children with acute otitis media. Front Genet.

[38] Farida H, Severin JA, Gasem MH, Keuter M, Wahyono H (2014). Nasopharyngeal carriage of *streptococcus pneumonia* in pneumonia-prone age groups in semarang, Java Island, Indonesia. PLoS One.

[39] Quirk SJ, Haraldsson G, Erlendsdóttir H, Hjálmarsdóttir MÁ, van Tonder AJ (2018). Effect of vaccination on pneumococci isolated from the nasopharynx of healthy children and the middle ear of children with otitis media in Iceland. J Clin Microbiol.

[40] Allemann A, Frey PM, Brugger SD, Hilty M (2017). Pneumococcal carriage and serotype variation before and after introduction of pneumococcal conjugate vaccines in patients with acute otitis media in Switzerland. Vaccine.

[41] Rosenblut A, Napolitano C, Pereira A, Moreno C, Kolhe D (2017). Etiology of acute otitis media and serotype distribution of *Streptococcus pneumoniae* and Haemophilus influenzae in Chilean children. Medicine.

[42] Alonso M, Marimon JM, Ercibengoa M, Pérez-Yarza EG, Pérez-Trallero E (2013). Dynamics of *Streptococcus pneumoniae* serotypes causing acute otitis media isolated from children with spontaneous middle-ear drainage over a 12-year period (1999-2010) in a region of northern Spain. PLoS One.

